# Supplementary material for: The Small RNA Universe of Capitella teleta
Source: Front Mol Biosci. 2022 Feb 25;9:802814. doi: 10.3389/fmolb.2022.802814 (PMC8915122; doi:10.3389/fmolb.2022.802814)
Supplement: Supplementary file 1 [file DataSheet1.ZIP › Supplement/known/CAPTEscaffold_421_20771.pdf]

Provisional ID	:	CAPTEscaffold_421_20771
Score total	:	23106.4
Score for star read(s)	:	3.9
Score for read counts	:	23097.8
Score for mfe	:	0.2
Score for randfold	:	1.6
Score for cons. seed	:	3
Total read count	:	45317
Mature read count	:	45009
Loop read count	:	0
Star read count	:	308

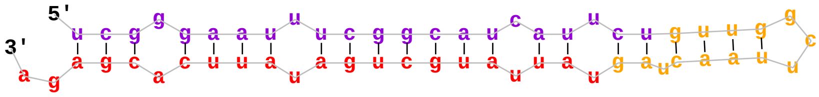

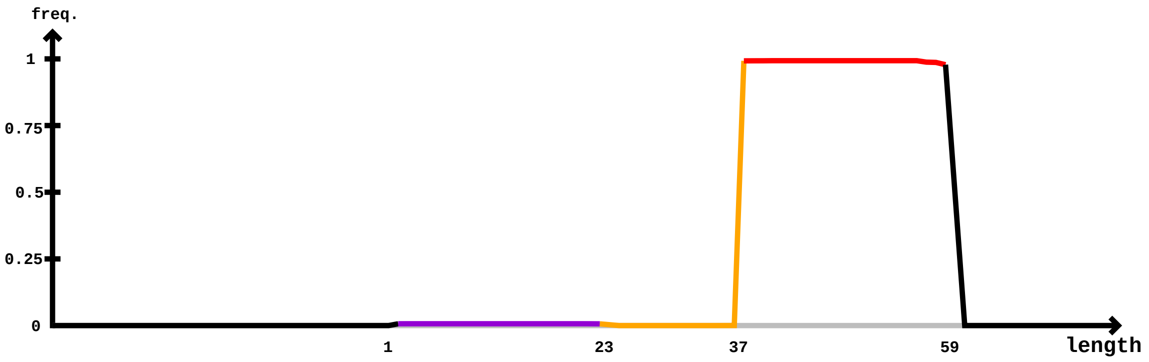

Star   Mature

```
5'- auggagauguuugaugauuauaaccucgugcauuucgggaauuucggcaucauucuguuggcuuaacuaguauuaugcugauauucacgagaugauuugaggacuuaca  -3'  obs
    auggagauguuugaugauuauaaccucgugcauuucgggaauuucggcaucauucuguuggcuuaacuaguauuaugcugauauucacgagaugauuugaggacuuaca       exp
    auggagauguuugaugauuauaaccucgugcauuucgggaauuucggcaucauucuguuggcuuaacuaguauuaugcugauauucacgagaugauuugaggacuuaca       known
    .......................(((((.((((((((.((((.(((((((.((.((((((...)))).)).)).))))))).)))).))))))).).))))).......   reads   mm   sample

    .................................uucgggaauuucggcaucauucu.....................................................   1       0    seq
    ..................................ucgggaauuucggcaucauu.......................................................   3       0    seq
    ..................................Acgggaauuucggcaucauuc......................................................   1       1    seq
    ..................................ucgggaauuucggcaucauuc......................................................   14      0    seq
    ..................................ucgggaauuuAggcaucauucu.....................................................   2       1    seq
    ..................................ucgggaauuucggcaucauucu.....................................................   245     0    seq
    ..................................ucgggaaAuucggcaucauucu.....................................................   1       1    seq
    ..................................ucgAgaauuucggcaucauucu.....................................................   2       1    seq
    ..................................Acgggaauuucggcaucauucu.....................................................   1       1    seq
    ..................................ucgggGauuucggcaucauucu.....................................................   1       1    seq
    ..................................uUgggaauuucggcaucauucu.....................................................   1       1    seq
    ..................................ucgggaauuucggcaucauucuA....................................................   1       1    seq
    ..................................ucgggaauuucggcaucauucug....................................................   29      0    seq
    ...................................cgggaauuucggcaucauucu.....................................................   3       0    seq
    ...................................cgggaauuucggcaucauucug....................................................   3       0    seq
    ...................................................................uGguauuaugcugauauucacgaga.................   1       1    seq
    ....................................................................Gguauuaugcugauauucacgaga.................   5       1    seq
    .....................................................................guauuaugcugauauucacg....................   2       0    seq
    .....................................................................guauuaugcugauauucacga...................   2       0    seq
    .....................................................................guauuaugcugauauucacgaga.................   1       0    seq
    ......................................................................uauuaugcugauauucGcg....................   1       1    seq
    ......................................................................Aauuaugcugauauucacg....................   1       1    seq
    ......................................................................uauuaugcuAauauucacg....................   1       1    seq
    ......................................................................uauuaugcugauauucacg....................   240     0    seq
    ......................................................................uauuaugcugauauucUcg....................   1       1    seq
    ......................................................................uauuaugcugauauucacgU...................   1       1    seq
    ......................................................................uauuaugcugauauucacga...................   44      0    seq
    ......................................................................uauuaCgcugauauucacgag..................   1       1    seq
    ......................................................................uauuGugcugauauucacgag..................   1       1    seq
    ......................................................................uauuaugcugauAucacgag..................   1       1    seq
    ......................................................................uauuaugcuAauauucacgag..................   1       1    seq
```

Star / Mature			
auggagauguuugaugauuauaaccucgugcauuucgggaauuucggcaucauucuguuggcuuaacuaguauuaugcugauauucacgagaugauuugaggacuuaca			
....uauuaAgcugauauucacgag..................	2	1	seq
....uaAuaugcugauauucacgag..................	2	1	seq
....uauuaugcugauauuAacgag..................	1	1	seq
....uauuaugcugauauucacgag..................	375	0	seq
....Aauuaugcugauauucacgag..................	1	1	seq
....uauuaugcugauauGcacgaga.................	1	1	seq
....uauuaugcugauauucacgaUa.................	7	1	seq
....Nauuaugcugauauucacgaga.................	14	1	seq
....uauuaugcugauauucGcgaga.................	18	1	seq
....uauuaugGugauauucacgaga.................	2	1	seq
....uauuaAgcugauauucacgaga.................	28	1	seq
....uauuaugcugauauucaGgaga.................	9	1	seq
....uGuuaugcugauauucacgaga.................	5	1	seq
....uauuaugcugauauAcacgaga.................	15	1	seq
....uauuaugcugauaAucacgaga.................	8	1	seq
....uauuaugcugauauucacgagU.................	11	1	seq
....uauuaugcugauauucacgaga.................	42200	0	seq
....uauuaugcugaAauucacgaga.................	12	1	seq
....uUuuaugcugauauucacgaga.................	1	1	seq
....uauuUugcugauauucacgaga.................	4	1	seq
....uauuaugcugauauuUacgaga.................	26	1	seq
....uauuaugcugauUuucacgaga.................	5	1	seq
....uauuaugcugauauucacAaga.................	140	1	seq
....uauuaugcAgauauucacgaga.................	6	1	seq
....uauuaugcugauauucCcgaga.................	1	1	seq
....uauuaugcugauauCcacgaga.................	3	1	seq
....uauuaugcuUauauucacgaga.................	5	1	seq
....uauuaugcugauauucacgaAa.................	7	1	seq
....uauuaugUugauauucacgaga.................	3	1	seq
....uauuaugcugauauucacgagG.................	45	1	seq
....uauCaugcugauauucacgaga.................	9	1	seq
....uauuaugcugauauucaAgaga.................	6	1	seq
....uauuaugcugauauuGacgaga.................	4	1	seq
....uauuaCgcugauauucacgaga.................	1	1	seq
....uauuaugAugauauucacgaga.................	10	1	seq
....Aauuaugcugauauucacgaga.................	181	1	seq
....uauuaugcGgauauucacgaga.................	2	1	seq
....uaAuaugcugauauucacgaga.................	48	1	seq
....uauuaugcugauauucacUaga.................	3	1	seq
....uauuaugcugUuauucacgaga.................	5	1	seq
....uauuaugcuCauauucacgaga.................	5	1	seq
....uauuaugcugauauuAacgaga.................	12	1	seq
....Gauuaugcugauauucacgaga.................	21	1	seq
....uauuaugcugauGuucacgaga.................	12	1	seq
....uauuaugcCgauauucacgaga.................	10	1	seq
....uauuaugcugauauucUcgaga.................	3	1	seq
....uauuaugcugaGauucacgaga.................	3	1	seq
....uauuauUcugauauucacgaga.................	13	1	seq
....uauuaugcugauauucaUgaga.................	6	1	seq
....uauuauCcugauauucacgaga.................	1	1	seq
....uauuaugcugaCauucacgaga.................	3	1	seq
....uauuaugcugauauucacgUga.................	4	1	seq
....uauuaugcugGuauucacgaga.................	7	1	seq
....uauuaugcugauaCucacgaga.................	3	1	seq
....uauuaugcuAauauucacgaga.................	44	1	seq
....uauuaugcugauauucacgGga.................	4	1	seq
....Cauuaugcugauauucacgaga.................	2	1	seq
....uauAaugcugauauucacgaga.................	10	1	seq
....uaCuaugcugauauucacgaga.................	5	1	seq
....uauuauAcugauauucacgaga.................	5	1	seq
....uauuGugcugauauucacgaga.................	7	1	seq
....uauuaugcugauaGucacgaga.................	2	1	seq
....uauuaugcugauauucacgaCa.................	1	1	seq
....uNuuaugcugauauucacgaga.................	1	1	seq
....uauuaugcugauUuucacgagau................	1	1	seq
....uauuaugcugauauucacgagau................	32	0	seq
....uauuaugcugauauucacgagaA................	1235	1	seq
....uauuaugcugauauucacgagaG................	1	1	seq
....uauuaugcugauauucacgagauU...............	1	1	seq
....auuaugcugauauucacgaga.................	4	0	seq

```
                                  Star                                  Mature
auggagauguuugaugauuauaaccucgugcauuucgggaauuucggcaucauucuguuggcuuaacuaguauuaugcugauauucacgagaugauuugaggacuuaca
.......................................................................auuaugcugauauucacgagaA................   5   1   seq
........................................................................uuaugcugauauucacgaga.................   2   0   seq
........................................................................uuaugcugauauucacgagaA................   1   1   seq
.........................................................................uaugcugauauucacgaga.................  14   0   seq
```
